# Supplementary material for: Empowering high-dimensional optical fiber communications with integrated photonic processors
Source: Nat Commun. 2024 Apr 25;15:3515. doi: 10.1038/s41467-024-47907-z (PMC11045856; doi:10.1038/s41467-024-47907-z)
Supplement: Supplementary file 1 — Supplementary Information [file 41467_2024_47907_MOESM1_ESM.pdf]

Supplementary Information for "Empowering high-dimensional optical fiber communications with integrated photonic processors"

Kaihang Lu[1, †], Zengqi Chen[1, †], Hao Chen[1, †], Wu Zhou[1], Zunyue Zhang[2, 3], Hon Ki Tsang[2, *], Yeyu Tong[1, *]

[1] Microelectronic Thrust, The Hong Kong University of Science and Technology (Guangzhou), 511453 Guangzhou, Guangdong, PR China

[2] Department of Electronic Engineering, The Chinese University of Hong Kong, Shatin, New Territories, 999077 Hong Kong, PR China

[3] School of Precision Instrument and Opto-Electronics Engineering, Tianjin University, 300072 Tianjin, PR China

[†] *These authors contributed equally to this work*

[*] *Corresponding author: hktsang@ee.cuhk.edu.hk, yeyutong@hkust-gz.edu.cn*

Contents

Supplementary Notes:

Note 1. Multimode optical I/O for few-mode fiber

Note 2. Mathematics and matrix operation of the integrated photonic processor

Note 3. Optical loss analysis of integrated photonic processor

Note 4. Chip-to-chip mode-division multiplexing optical communications

Supplementary Figures:

Figure S1. Design and optimization of the multimode optical I/O

Figure S2. Simulated chip-to-fiber and fiber-to-chip coupling efficiency

Figure S3. Integrated photonic processor at the receiver side

Figure S4. Experimental setup for mode-division multiplexing optical communications by the integrated photonic processor

Figure S5. Eye diagrams for chip-to-chip optical fiber communications using a single data lane encoded on different fiber LP modes

Supplementary Tables

Table S1. Comparison of integrated multimode optical I/O for multi-mode fibers

Note 1. Multimode optical I/O for few-mode fiber

In this work, a two-dimensional (2D) grating coupler is utilized as the multimode optical I/O to facilitate the conversion between the planar waveguide mode on the chip and the 2D field distribution of the optical fiber. Four linearly tapered mode size converters and four tapered asymmetrical directional couplers (ADCs)[1,2] are employed to convert between all the planar waveguide modes and eight fundamental quasi-transverse-electric (TE) modes prior to the optical mesh, as illustrated by Figure S1a.

Selective fiber mode launching at the transmitter can be realized via controlling the relative phase difference between the two counterpropagating TE modes. Since the fundamental (TE_0) and first-order (TE_1) modes exhibit similar effective indices in the silicon grating region with a width of 13 μm, they can be efficiently diffracted out of the plane relying on the same grating structure. The linearly polarized (LP) modes in a two-mode few-mode fiber (FMF) include $LP_{01\text{-}x}$, $LP_{01\text{-}y}$, $LP_{11a\text{-}x}$, $LP_{11a\text{-}y}$, $LP_{11b\text{-}x}$, and $LP_{11b\text{-}y}$. The corresponding launching conditions for each optical fiber mode are summarized in Figure S1b.

The 2D grating coupler, with a width and length of 13.0 μm, is specially designed to match the mode field diameter of 11.0 μm for the LP_{01} and LP_{11} modes in the two-mode graded-index few-mode fiber (FMF) from *OFS*. The width of silicon waveguide is linearly tapered from 0.962 μm to 13.0 μm by an adiabatic linear taper with a length of L_{taper}=350 μm as shown in Figure S1a. Figure S1c illustrates the schematic of the tapered ADC for (de)multiplexing the TE_0-TE_1 mode on-chip. The integrated waveguide width w_1, w_{2a}, and w_{2b} are 0.45 μm, 0.902 μm, and 0.962 μm, respectively. The waveguide gap g is designed as 0.2 μm and coupling length L is 33.6 μm.

The 2D grating coupler is formed by 70-nm shallowly etched circular holes, as depicted in Figure S1d.The grating diffraction region is centrosymmetric to ensure uniform coupling performance for all optical signals from the four orthogonally positioned waveguides. To achieve efficient and fully vertical coupling, the grating periods are chirped and optimized using a genetic optimization algorithm [3] and finite-difference time domain (FDTD) simulations.

The optimization process evaluates coupling efficiency of the grating coupler using effective medium theory [4,5] and 2D FDTD simulation. This is advantageous as each simulation can be completed in just a few seconds, made possible by two key factors. Firstly, the grating region is symmetrical for the two polarizations, allowing for the consideration of only one polarization during optimization. Additionally, mode TE_0 and TE_1 exhibit very similar effective indices in the 13.0 μm wide waveguide. As a result, the coupling efficiency of the fundamental TE_0 mode in a single polarization can serve as the figure of merit in the optimization iteration loop. The genetic optimization algorithm is utilized to optimize both the grating period and circular hole diameter. The optimal hole diameter is 343 nm. The chirped grating periods are depicted in Figure S1e. Figure S1f illustrates the evolution diagram of coupling efficiency during the optimization process using genetic algorithm.

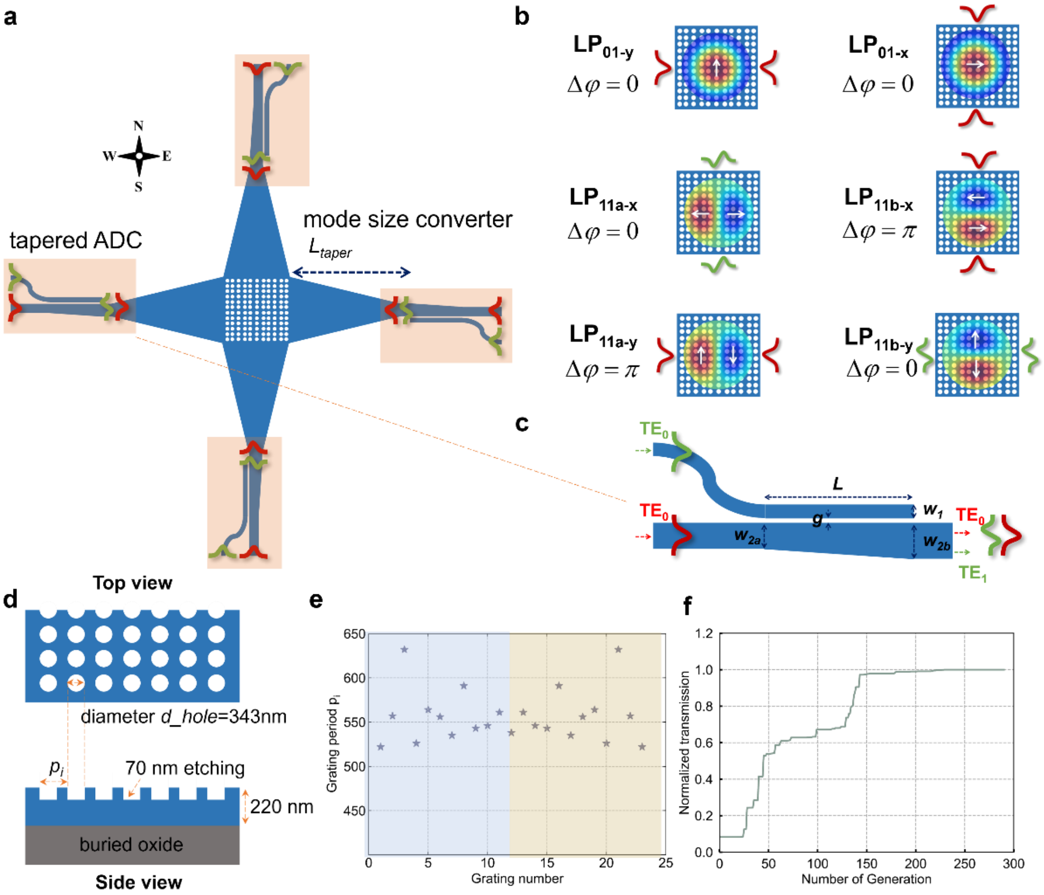

Figure S1 Design and optimization of the multimode optical I/O. **a** Schematic of multimode optical I/O, consisting of a two-dimensional (2D) waveguide grating, four linearly tapered mode size converters, and four tapered asymmetrical directional couplers (ADCs). **b** Illustration of fiber mode (de)multiplexing using the multimode grating coupler. **c** Schematic of the tapered ADC for TE_0-TE_1 mode (de)multiplexing. **d** Top view and

side view of the 2D grating coupler. **e** Optimal grating periods obtained by genetic optimization. The symmetrical grating period is shown by the transparent windows. **f** Evolution diagram of normalized coupling efficiency during the optimization process.

The optimal grating coupler performance is verified by 3D-FDTD simulation before fabrication. Figure S2a depicts the simulated spectra of the chip-to-fiber coupling efficiency for the 2D multimode grating coupler. The x-polarized LP_{01}, LP_{11a}, and LP_{11b} modes manifest a similar coupling efficiency of about –4.6 dB at a wavelength of 1558 nm. The wavelength span is about 21 nm with a mode-dependent loss is less than 3 dB. At the receiver side, because of random mode evolution and polarization rotation in a circular core FMF, the received speckle pattern is uncertain at the fiber end. However, the grating coupler can support efficient coupling for all the eigenmodes in a two-mode FMF. To validate that, fiber-to-chip coupling simulation is performed by launching various eigenmodes in a two-mode FMF and summing the received optical power into TE_0 and TE_1 modes on chip. The coupling loss spectra for $HE_{11\text{-}x}$, $HE_{11\text{-}y}$, TM_{01}, TE_{01}, $HE_{21\text{-even}}$, and $HE_{21\text{-odd}}$ as shown in Figure S2b. Equation S1-S4 illustrate the corresponding relationship between the fiber eigen modes and the LP_{11} mode. The two simulation results in Figure S2 show good agreement with a minor discrepancy, suggesting that the multimode optical I/O can be utilized at both the transmitter and receiver sides, with small mode-dependent loss.

$$\mathrm{LP}_{11\text{a-x}} = \mathrm{TM}_{01} + e^{j\pi}\mathrm{HE}_{21}{}^{\text{even}} \tag{S1}$$

$$\mathrm{LP}_{11\text{a-y}} = \mathrm{TM}_{01} + \mathrm{HE}_{21}{}^{\text{odd}} \tag{S2}$$

$$\mathrm{LP}_{11\text{b-x}} = \mathrm{TE}_{01} + e^{j\pi}\mathrm{HE}_{21}{}^{\text{odd}} \tag{S3}$$

$$\mathrm{LP}_{11\text{b-y}} = \mathrm{TM}_{01} + \mathrm{HE}_{21}{}^{\text{even}} \tag{S4}$$

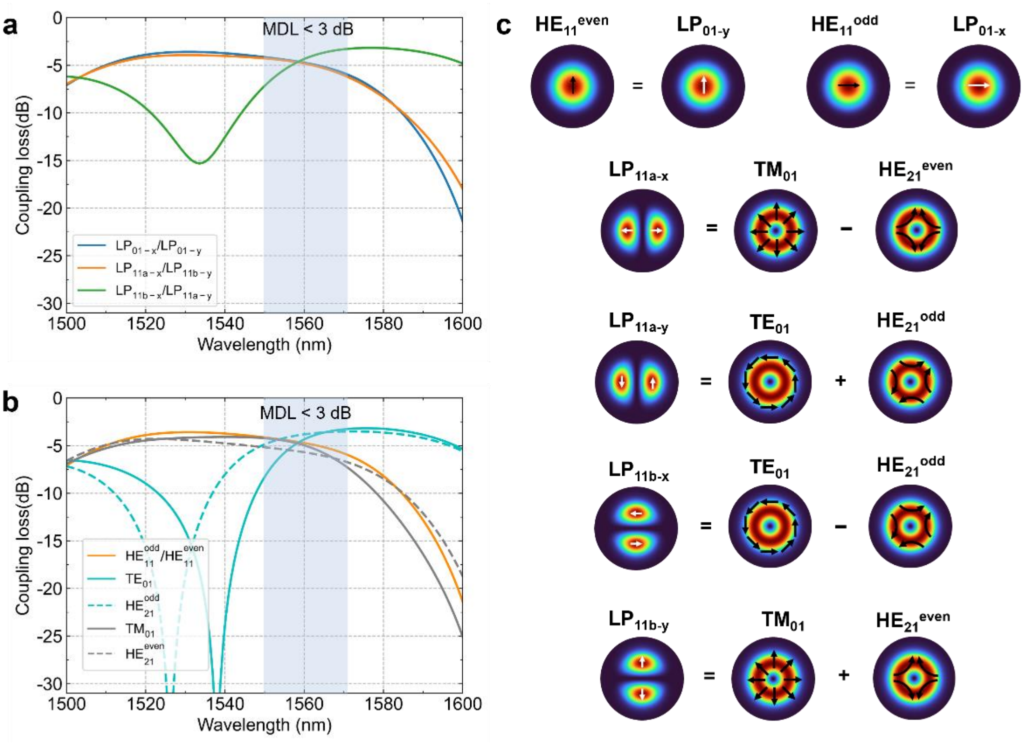

Figure S2. Simulated chip-to-fiber and fiber-to-chip coupling efficiency. a Chip-to-fiber coupling efficiency spectra by launching various LP modes in a two-mode FMF at the transmitter side. The two orthogonal polarizations exhibit similar results because of the structure symmetry. **b** Fiber-to-chip coupling efficiency spectra by launching various eigenmodes in a two-mode FMF at the receiver side. **c** Correspondence between the LP modes and eigenmodes in a two-mode FMF.

Table S1. Comparison of integrated multimode optical I/O for multi-mode fibers

Design	Fiber type	Num. of spatial channels	Experimental coupling efficiency [dB]
Grating array[6]	2-mode circular core FMF	6	LP_{01}-LP_{11}: <−20
Grating array[7]	2-mode circular core FMF	6	LP_{01}-LP_{11}: −23
2D grating[8]	2-mode circular core FMF	8	LP_{01}: −22
2D grating[9]	2-mode circular core FMF	4	LP_{01}: −4.9, LP_{11}: −6.1
Grating coupler[10]	2-mode circular core FMF	2	LP_{01}: −1.36, LP_{11}: −2.21
Grating array[11]	2-mode circular core FMF	6	LP_{01}: −5.2, LP_{11}: −9.0
Edge coupler[12]	2-mode circular core FMF	2	LP_{01}: −13.2, LP_{11}: −12.5
2D grating This work	2-mode circular core FMF	6	LP_{01}: −3.5, LP_{11}: −6.1

Note 2. Mathematics and matrix operation of the integrated photonic processor

In this section, the matrix operation from the transmitter side to the received side is explained. Based on the low-loss and multimode grating coupler design illustrated in Note 1, the integrated photonic processor adopts an 8-dimensional matrix transformation after the multimode optical I/O to retrieve the 6 orthogonal channels allowed in a two-mode FMF. The multimode optical I/O at the receiver side can non-selectively convert the six eigenmodes in a two-mode FMF into the eight single-mode channels on chip. Therefore, the transmission matrix of the multimode grating coupler at the receiver side can be expressed as an 8 by 6 matrix A_{8x6}, as denoted by Equation S5. The corresponding optical signal distribution for each fiber eigenmode after the multimode grating coupler can be explained by Figure S3. The transfer matrix of multimode grating coupler at the transmitter side is expressed as pinv*(A)*, which is the pseudo-inverse of the transfer matrix A.

$$A = \begin{pmatrix} \frac{\sqrt{2}}{2} & 0 & \frac{1}{2} & \frac{1}{2} & 0 & 0 \\ 0 & 0 & 0 & 0 & -\frac{1}{2} & \frac{1}{2} \\ 0 & \frac{\sqrt{2}}{2} & \frac{1}{2} & -\frac{1}{2} & 0 & 0 \\ 0 & 0 & 0 & 0 & \frac{1}{2} & \frac{1}{2} \\ \frac{\sqrt{2}}{2} & 0 & -\frac{1}{2} & -\frac{1}{2} & 0 & 0 \\ 0 & 0 & 0 & 0 & -\frac{1}{2} & \frac{1}{2} \\ 0 & \frac{\sqrt{2}}{2} & -\frac{1}{2} & \frac{1}{2} & 0 & 0 \\ 0 & 0 & 0 & 0 & \frac{1}{2} & \frac{1}{2} \end{pmatrix} \tag{S5}$$

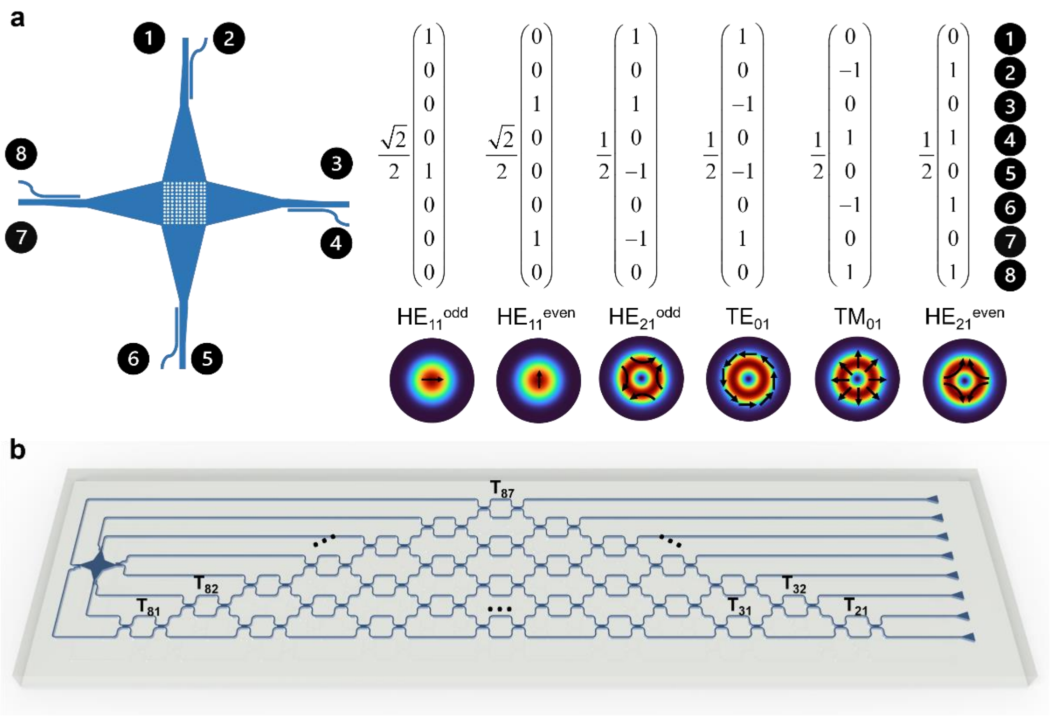

Figure S3. Integrated photonic processor at the receiver side. a Optical field distributions in the eight single-mode waveguides on chip for each eigenmode supported in a two-mode FMF. **b** Schematic of the 8×8 unitary optical mesh *U(8)* based on a triangular array of 28 tunable 2×2 beam splitters.

The speckle pattern arriving at fiber end of the receiver side can always be decomposed into a linear combination of all the eigenmodes supported in a two-mode FMF due to completeness, as denoted as:

$$E_{rx} = \sum_{n=1}^{N} c_n E_n \tag{S6}$$

where N represents the total number of the supported eigenmodes of the FMF, c_n denotes the weighting coefficient and phase state of each guided eigenmode.

The photonic integrated processor at the receiver side employs an 8×8 unitary optical mesh *U(8)* to undo the unknown and arbitrary signal mixing among the complete set of 6 orthogonal channels inside of a two-mode FMF. The transformation matrix M from the transmitter side to the receiver side can thus be expressed as:

$$M = U_{8\times8} A_{8\times6} U_{6\times6} \,\mathrm{pinv}(A)_{6\times8} \tag{S7}$$

where *U(6)* denote the transformation matrix of the two-mode FMF. The unitary optical mesh is composed of a triangular array of 28 tunable 2×2 beam splitters (BSs). As shown in Figure

S3, each of beam splitters forms simple 2×2 unitary transformations consisting of two heater-based optical phase shifters and two multimode interferometers (MMIs). The corresponding scattering matrix of each BS can be obtained from the product of the subcomponents shown by Equation S8, where θ_1 and θ_2 are the outer and inner optical phase shifts obtained by the tunable heaters shown in Figure 2e. Equation S8 can be simplified to Equation S9 by applying Euler's equation. Unitary transformation *U(2)* can thus be realized as indicated by Equation S10. An 8×8 unitary transformation matrix can be formed by cascading the tunable BSs in a triangular topology as depicted by Equation S11.

$$S = \begin{pmatrix} \frac{1}{\sqrt{2}} & \frac{j}{\sqrt{2}} \\ \frac{j}{\sqrt{2}} & \frac{1}{\sqrt{2}} \end{pmatrix} \begin{pmatrix} e^{j\theta_2} & 0 \\ 0 & 1 \end{pmatrix} \begin{pmatrix} \frac{1}{\sqrt{2}} & \frac{j}{\sqrt{2}} \\ \frac{j}{\sqrt{2}} & \frac{1}{\sqrt{2}} \end{pmatrix} \begin{pmatrix} e^{j\theta_1} & 0 \\ 0 & 1 \end{pmatrix} \tag{S8}$$

$$S = je^{j\frac{\theta_2}{2}} \begin{pmatrix} e^{j\theta_1}\sin\frac{\theta_2}{2} & \cos\frac{\theta_2}{2} \\ e^{j\theta_1}\cos\frac{\theta_2}{2} & -\sin\frac{\theta_2}{2} \end{pmatrix} \tag{S9}$$

$$SS^{\dagger} = S^{\dagger}S = E \tag{S10}$$

$$U(N) = \prod_{i=2}^{N}\left[\prod_{j=i-1}^{1} T_{ij}\right] \tag{S11}$$

where T_{ij} is shown in Equation S12, i is from 2 to N, $j = i - 1$. Each BS changes a two-dimensional subspace of Equation S11.

$$T_{ij} = \begin{pmatrix} 1 & 0 & \cdots & \cdots & \cdots & \cdots & 0 \\ 0 & 1 & \cdots & \cdots & \cdots & \cdots & 0 \\ \vdots & \vdots & \ddots & \cdots & \cdots & \cdots & \vdots \\ 0 & 0 & \cdots & e^{j\theta_1}\sin\frac{\theta_2}{2} & \cos\frac{\theta_2}{2} & \cdots & 0 \\ 0 & 0 & \cdots & e^{j\theta_1}\cos\frac{\theta_2}{2} & -\sin\frac{\theta_2}{2} & \cdots & 0 \\ \vdots & \vdots & \cdots & \cdots & \cdots & \ddots & \vdots \\ 0 & 0 & \cdots & \cdots & \cdots & \cdots & 1 \end{pmatrix} \tag{S12}$$

Note 3. Optical loss analysis of integrated photonic processor

In the system experiment, chip-to-chip high-speed optical communications are conducted at a wavelength of 1530 nm. This choice of wavelength is primarily motivated by three reasons: Firstly, the single-mode grating array exhibits a center wavelength of 1535 nm with a coupling loss of –4.5 dB, while the coupling loss increases to –8 dB at 1560 nm. Secondly, the multimode grating coupler demonstrates low mode-dependent loss at 1530 nm. Lastly, 1530 nm falls within the working wavelength range of the erbium-doped fiber amplifier (EDFA) for optical amplification to compensate for optical power loss. In the future, the center wavelength of all optical I/O devices and the optical mesh can be aligned to optimize the overall loss of the communication system.

At 1530 nm, the optimal insertion loss of the transmitter side is approximately 10 dB, with a loss variation of roughly 3 dB for all spatial channels. An external tunable laser serves as the light source. At the transmitter side, each single-mode input grating coupler experiences a transmission loss of approximately 5 dB. The multimode grating coupler has a coupling loss of about 4~7 dB for LP_{01} and LP_{11} mode groups, respectively. In the future, the insertion loss and loss variation can be improved by optimizing the center wavelength of the input single-mode grating coupler and multimode grating coupler.

At 1530 nm, the optimal total insertion loss of the receiver side is approximately 15 dB, with a loss variation within 6 dB due to different routing paths with varying numbers of MZIs and the mode-dependent loss of the multimode grating coupler. At the receiver side, the optical loss can be broken down into 4~7 dB from the multimode grating coupler, 5 dB from the single mode output grating coupler, around 0.35 dB for each multimode interferometer (MMI) (center wavelength is designed at 1550 nm), about 4.5 dB from the waveguide and bends inside the optical mesh. In the future, the total loss and loss variation can be mitigated by aligning the center working wavelength of the MZIs to the multimode grating coupler.

Note 4. Chip-to-chip mode-division multiplexing optical communications

A chip-to-chip high-dimensional optical communication system is established by the integrated photonic processor. Figure S4a presents the experimental setup for the high-speed eye diagram

measurements when different fiber modes are selectively launched from the transmitter side and descrambled by the integrated optical mesh at the receiver side after the configuration. We also evaluate the mode descrambling performance at the receiver side with additional orthogonal modes turned on. Since the input grating coupler array of the integrated transmitter is not compatible with a fiber array unit with channel pitch of 250 μm, a fiber-based mode selective photonic lantern [13] is utilized as shown in Figure S4c-S4d. Two concurrent channels are launched and decoupled by using the experimental setup shown in Figure S4c. The combination of LP_{01} with LP_{11a}, LP_{01} with LP_{11b}, and LP_{11a} with LP_{11b} are evaluated, as indicated by the blue, green, and red lines in the dotted box. The optical mesh is configured to decouple the two orthogonal modes. Eye diagram and bit error rates are measured when the two current data lanes are launched from the transmitter side. To evaluate concurrent injection of three modes, LP_{01}, LP_{11a}, and LP_{11b} modes are spatially decoupled first by 2-km and 5-km SMFs before launching into the fiber photonic lantern. The photonic processor at the receiver side will be configured to reconstruct high-speed eye diagrams for one of the spatial channels. Once completed, the other two spatial channels will be activated to assess the induced penalty on the eye diagrams and bit error rates.

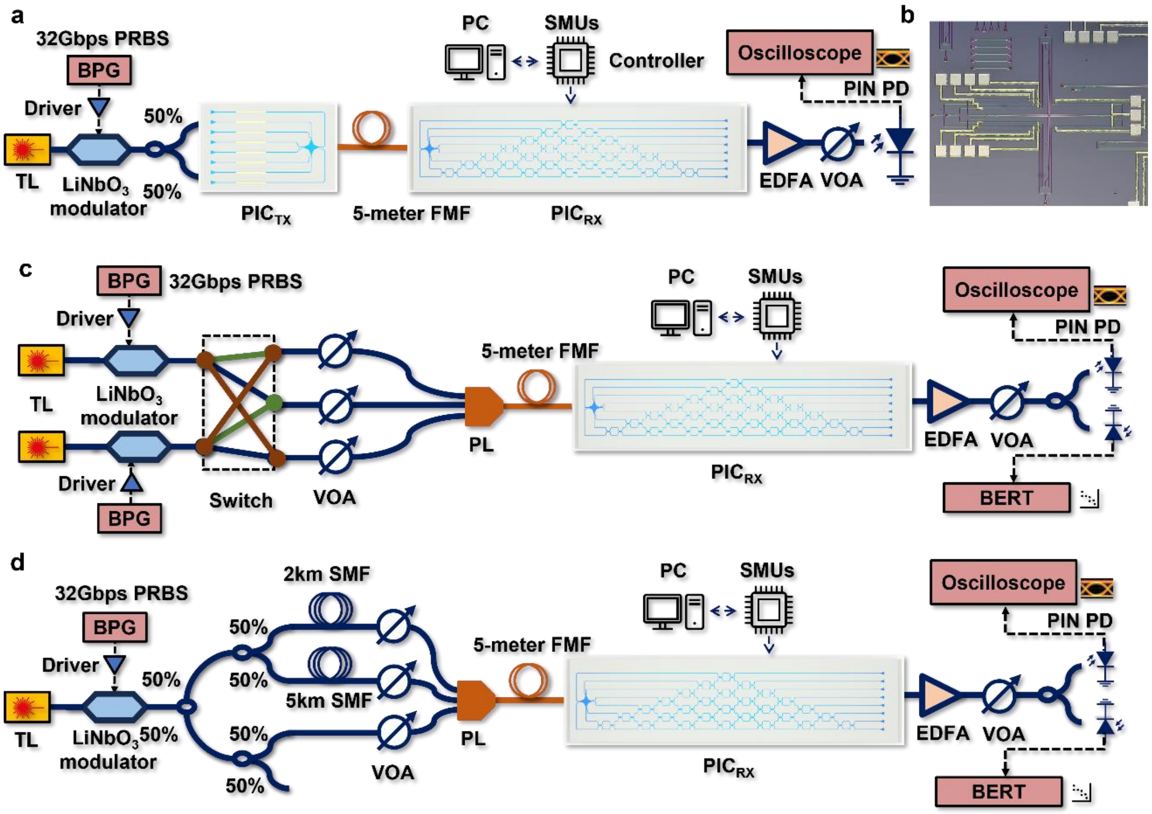

Figure S4. Experimental setup for mode-division multiplexing optical communications by the integrated photonic processor. a Experimental setup for inter-chip optical communications using a single data lane encoded on various fiber LP modes. **b** Microscopic image of the photonic chip at the transmitter side for selective mode launching. **c** Experimental setup for MDM optical fiber communications by simultaneous launching of two LP modes from the transmitter side. Blue, green, and red lines in the dotted box represent three different combinations. **d** Experimental setup for evaluation of all-optical mode descrambling with three spatially decoupled fiber modes including LP$_{01}$, LP$_{11a}$, and LP$_{11b}$ injected simultaneously. TL: tunable laser; BPG: bit pattern generator; SMF: standard single mode fiber; FMF: few-mode fiber; PC: personal computer; SMUs: multichannel source-measurement units; EDFA: erbium-doped fiber amplifier; VOA: variable optical attenuator; PD: photodiode; PL: mode-selective photonic lantern. PRBS: pseudorandom binary sequence; BERT: bit error rate tester; PIC$_{TX}$: photonic integrated circuits at the transmitter side; PIC$_{RX}$: photonic integrated circuits at the receiver side.

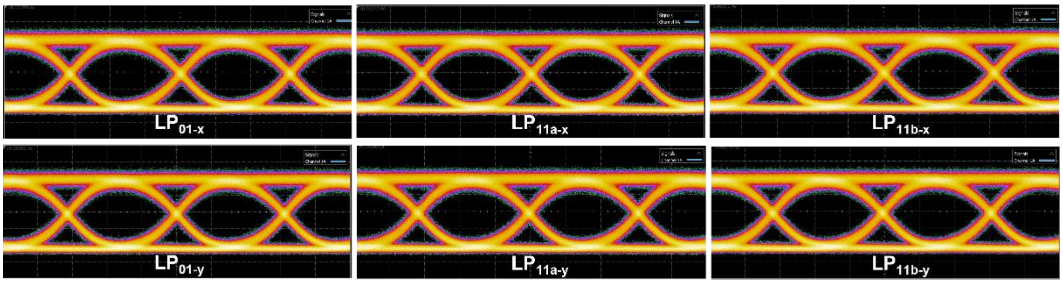

Figure S5. Eye diagrams for chip-to-chip optical fiber communications using a single data lane encoded on different fiber LP modes. 32Gbps eye diagrams of the six spatial and polarization channels launched and retrieved by the integrated photonic processor for mode LP$_{01-x}$, LP$_{01-y}$, LP$_{11a-x}$, LP$_{11a-y}$, LP$_{11b-x}$, LP$_{11b-y}$.

Supplementary References

1. Ding, Y. *et al.* On-chip two-mode division multiplexing using tapered directional coupler-based mode multiplexer and demultiplexer. *Optics Express* **21**, 10376–10382 (2013).
2. Daoxin, D., Jian, W. & Yaocheng, S. Silicon mode (de)multiplexer enabling high capacity photonic networks-on-chip with a single-wavelength-carrier light. *Optics Letters* **38**, 1422–1424 (2013).
3. Mitchell, M. *An Introduction to Genetic Algorithms*. (MIT Press, Cambridge, MA, USA, 1998).
4. RYTOV, S. Electromagnetic properties of a finely stratified medium. *Soviet Physics JEPT* **2**, 466–475 (1956).
5. Chen, X. & Tsang, H. K. Nanoholes Grating Couplers for Coupling Between Silicon-on-Insulator Waveguides and Optical Fibers. *IEEE Photonics Journal* **1**, 184–190 (2009).
6. Koonen, A. M. J., Chen, H., Boom, H. P. A. van den & Raz, O. Silicon Photonic Integrated Mode Multiplexer and Demultiplexer. *IEEE Photonics Technology Letters* **24**, 1961–1964 (2012).
7. Ding, Y., Ou, H., Xu, J. & Peucheret, C. Silicon Photonic Integrated Circuit Mode Multiplexer. *IEEE Photon. Technol. Lett.* **25**, 648–651 (2013).
8. Wohlfeil, B. *et al.* A Two-Dimensional Fiber Grating Coupler on SOI for Mode Division Multiplexing. *IEEE Photonics Technology Letters* **28**, 1241–1244 (2016).
9. Tong, Y., Zhou, W., Wu, X. & Tsang, H. K. Efficient Mode Multiplexer for Few-Mode Fibers Using Integrated Silicon-on-Insulator Waveguide Grating Coupler. *IEEE Journal of Quantum Electronics* **56**, (2019).
10. Zhou, X. & Tsang, H. K. High Efficiency Multimode Waveguide Grating Coupler for Few-Mode Fibers. *IEEE Photonics J.* **14**, 1–5 (2022).
11. Watanabe, T. *et al.* Coherent few mode demultiplexer realized as a 2D grating coupler array in silicon. *Opt. Express* **28**, 36009 (2020).
12. Shen, W., Du, J., Xiong, J., Ma, L. & He, Z. Silicon-integrated dual-mode fiber-to-chip edge coupler for 2 × 100 Gbps/lambda MDM optical interconnection. *Opt. Express, OE* **28**, 33254–33262 (2020).
13. Leon-Saval, S. G. *et al.* Mode-selective photonic lanterns for space-division multiplexing. *Optics Express* **22**, 1036–1044 (2014).
